# Epidemiology and Genetic Traits of Carbapenemase-Producing Enterobacterales: A Global Threat to Human Health

**DOI:** 10.3390/antibiotics14020141

**Published:** 2025-02-01

**Authors:** Gualtiero Alvisi, Antonio Curtoni, Rossella Fonnesu, Aurora Piazza, Caterina Signoretto, Giorgia Piccinini, Davide Sassera, Paolo Gaibani

**Affiliations:** 1 Department of Molecular Medicine, University of Padua, 35135 Padova, Italy; gualtiero.alvisi@unipd.it; 2 Department of Public Health and Paediatrics, University of Turin, 10100 Turin, Italy; antonio.curtoni@unito.it (A.C.); giorgia.piccinini@unito.it (G.P.); 3 Microbiology and Virology Unit, University Hospital Città della Salute e della Scienza di Torino, 10100 Turin, Italy; 4 Microbiology and Virology Unit, Azienda Ospedaliera Universitaria Integrata Di Verona, 37134 Verona, Italy; rossella.fonnesu@aovr.veneto.it (R.F.); caterina.signoretto@univr.it (C.S.); 5 Department of Clinical Surgical Diagnostic and Pediatric Sciences, University of Pavia, 27100 Pavia, Italy; aurora.piazza@unipv.it; 6 Unit of Microbiology and Clinical Microbiology, Department of Clinical-Surgical, Diagnostic and Pediatric Sciences, University of Pavia, 27100 Pavia, Italy; 7 Department of Diagnostics and Public Health, Microbiology Section, Verona University, 37134 Verona, Italy; 8 PhD National Programme in One Health Approaches to Infectious Diseases and Life Science Research, Department of Public Health, Experimental and Forensic Medicine, University of Pavia, 27100 Pavia, Italy; 9 Department of Biology and Biotechnology, University of Pavia, 27100 Pavia, Italy; davide.sassera@unipv.it; 10 Fondazione IRCCS Policlinico San Matteo, 27100 Pavia, Italy

**Keywords:** carbapenemase, antimicrobial resistance, epidemiology, KPC, NDM, VIM, IMP, OXA-48

## Abstract

Carbapenemase-producing Enterobacterales (CPE) represent an important threat to global health, resulting in an urgent issue in clinical settings. CPE often exhibit a multidrug-resistant (MDR) phenotype, thus reducing the antimicrobial armamentarium, with few antibiotics retaining residual antimicrobial activity against these pathogens. Carbapenemases are divided into three classes (A, B, and D) according to the Ambler classification system. Among these, KPC (class A), NDM, VIM, IMP (class B), and OXA-48-like (class D) represent the most important carbapenemases in terms of diffusion and clinical impact. CPE diffusion has been observed worldwide, with current endemicity in multiple territories around the world. In this context, the clonal spread and plasmid-mediated transmission of carbapenemases have contributed to the global spread of CPE worldwide and to the diffusion of carbapenemases among different Enterobacterales species. In recent years, novel molecules showing excellent in vitro and in vivo activity have been developed against CPE. However, the recent emergence of novel traits of resistance to these molecules has already been reported in several cases, mitigating the initial promising results. This review aims to provide an updated description of the major classes of carbapenemases, their global distribution, and future perspectives to limit the diffusion of CPEs.

## 1. Introduction

Carbapenems represent a category of antibiotics that are often used as a last-line option for the treatment of multidrug-resistant (MDR) Gram-negative bacterial infections [1,2,3,4,5,6]. Bacteria classified as Carbapenemase-producing Enterobacterales (CPE) are endowed with the ability to hydrolyze carbapenems, thereby eluding carbapenems’ antimicrobial activity. Due to the importance of carbapenems in fighting MDR bacterial infections, the spread of CPE represents a major issue worldwide, further limiting available therapeutic options [7,8].

The term “carbapenemase” refers to a wide range of enzymes produced by bacteria conferring resistance to carbapenems. Among these, the best-characterized carbapenemases are *Klebsiella pneumoniae* carbapenemase (KPC), New Delhi metallo-ß-lactamase (NDM), and oxacillinase-48 (OXA-48)-like enzymes [1,2,3,4]. Each of these enzymes is characterized by peculiar genetic and biochemical properties that influence their epidemiology and therapeutic response (Table 1). Although the ability to produce KPC was initially described primarily for Enterobacterales, it has been subsequently found in different bacterial species, including *Acinetobacter* and *Pseudomonas* [3,4]. Similarly, travel and international trade have promoted the spread of NDM-producing bacteria across the globe [5]. Along the same lines, OXA-48 producers have widely spread across European countries, especially in the Mediterranean region, with ongoing reports of hyperendemicity [6,7].

The epidemiology of CPE reflects a complex interplay of environmental factors, healthcare practices, and individual behaviors and varies significantly across geographical regions, as it is influenced by local antibiotic usage patterns, infection control measures, and healthcare infrastructure quality. For example, high rates of CPE prevalence have been reported in Europe and Latin America, where most infections occur within healthcare settings [7]. The World Health Organization (WHO) has recognized carbapenem-resistant Enterobacterales as critical priority pathogens due to their rising prevalence and associated public health risks [8], which highlights the urgent need for effective surveillance and containment strategies [9].

Moreover, the transmission dynamics of CPE infections often involve hospital outbreaks. Such a situation is the result of three main factors. First of all, hospitals represent a formidable and vast reservoir of commensal Enterobacterales harboring carbapenemase genes due to asymptomatic carriers among both healthcare workers and patients. Second, the presence of patients with prolonged hospitalization strongly increases the risk of infection. Finally, the presence of invasive devices and prior antibiotic exposure further exacerbates the situation, facilitating the spread of CPE among hospitalized individuals [2,3].

Due to the impact that CPE infections have on patient outcomes and healthcare systems, their management is becoming increasingly important [8]. Traditional antibiotics are often ineffective against CPE, leading to a plethora of consequences, such as prolonged hospitalizations, as well as increased morbidity and mortality rates. Indeed, infections with CPE have been linked to mortality rates exceeding 50%, particularly in critically ill patients [1,2]. Therefore, developing effective treatments for CPE infections has emerged as an important goal of antimicrobial research [8]. A few traditional agents, such as aminoglycosides and polymyxins, have shown activity against some CPE; however, their usage has been strongly limited by dosing regimens, high toxicity, and the rapid emergence of resistance [2]. Recent advances have been the development of newer β-lactam/β-lactamase inhibitor combinations, such as ceftazidime-avibactam (CAZ-AVI) and meropenem-vaborbactam (MER-VAB), which have proven efficient against several CPE (Table 2) [10]. Unfortunately, resistance to these novel agents further complicates the therapeutic landscape, revealing the urgent need for ongoing research and development of alternative antibiotics [11,12].

The implementation of effective infection control measures appears to be easier to achieve compared to the development of new therapeutic regimens, but its impact should not be underestimated [8]. Indeed, strict adherence to hygiene practices, the appropriate use of personal protective equipment, and comprehensive surveillance programs could strongly reduce the transmission of CPE within healthcare settings. Patient screening and isolation protocols are critical components of infection control strategies that aim to limit the spread of resistant organisms. Moreover, education and training programs for healthcare professionals would raise awareness surrounding antibiotic stewardship and infection prevention [13]. While important advances have been made toward understanding the epidemiology of CPE and developing targeted therapeutic strategies, the fight against antibiotic resistance requires a multifaceted approach. Initiatives aimed at improving surveillance, enhancing antimicrobial stewardship, reducing unnecessary antibiotic prescriptions, and promoting responsible antibiotic use will be crucial in this respect [14]. Along the same lines, initiatives aimed at reducing the overuse of antibiotics in agriculture, combined with improved hygiene practices in animal husbandry, must also be implemented [15].

Furthermore, governmental and institutional funding for research into novel antibiotics and alternative therapies must be increased to mitigate the risks associated with the spread of CPE. Among these, phage therapy, immunotherapy, and the development of rapid diagnostic tests will likely play a pivotal role in controlling the CPE clinical burden.

In this review, we summarize the classification of the different types of carbapenemases and the epidemiology of the principal carbapenemases in different countries.

## 2. Classification of Carbapenamases in Enterobacterales

β-lactamases classification was historically founded on two systems: the Bush–Jacoby–Medeiros system, which is based on the enzyme biochemical activity, and Ambler’s system, which is based on the enzyme molecular structure [16,17,18,19].

The most widely used system, Ambler classification, categorizes carbapenemases into two major groups: serine-β-lactamases (SBL), which are characterized by the presence of a serine in the enzyme active site, and metallo-β-lactamases (MBL), which have a bivalent metal cation, usually *Zn* in the catalytic site [20]. According to the amino acid sequence homology, carbapenemases could be further divided into different classes: A and D, belonging to the SBL group, and B, belonging to the MBL group [20]. In addition to the chemical structures, among these classes, different mechanisms of action, substrate spectra, catalytic efficiencies, and inhibitors can be identified (Table 1).

### 2.1. Class A Carbapenemases

In class A carbapenemases, the serine residue in position 70 of the catalytic site is pivotal for hydrolytic activity. As part of the Bush–Jacoby–Medeiros functional group 2, they are able to hydrolyze a wide variety of β-lactams, such as penicillins, cephalosporins, monobactams, and carbapenems. They are inactivated by boronic acid derivates and partially inactivated by β-lactamase inhibitors, such as tazobactam and, more weakly, clavulanic acid [21].

The major families included in this class are the NMC, IMI, SME, SHV-38, SFC-1, FPH-1, PenA, GES, and KPC enzymes [21,22,23,24,25,26,27]. With the notable exception of KPC, most of them are usually chromosomally encoded, rarely associated with Enterobacterales, or of limited clinical interest, with sporadic cases and outbreaks.

#### KPC

Among class A, KPC, which is an acronym for *K. pneumoniae* carbapenemase, is the most important enzyme, and it is listed among the “big five” carbapenemases. First isolated in North Carolina in 1996 thanks to its ability to move via horizontal gene transfer through plasmids, KPC disseminated across Enterobacterales and became a major global health threat [21]. Its worldwide spread was principally linked to *K. pneumoniae,* in particular, ST258 and, more recently, ST512 and mobile genetic elements such as *Tn3*-based *Tn4401* transposon [28,29,30]. Other Enterobacterales that more rarely harbor the KPC enzyme include *Escherichia coli*, *Citrobacter*, *Enterobacter*, *Serratia*, *Proteus*, and *Morganella*.

KPC distribution varies between the continents, with KPC-2 and KPC-3 being predominant; however, to date, more than 150 KPC variants have been identified [30,31].

KPC enzymes have a molecular weight of about 32 kDa, and their structure is similar to that of other class A β-lactamases and contains two subdomains: one α-helical and five β-strands surrounded by α-helices [31,32]. The active site is located at the interface between the two domains, with the serine residue (Ser70), surrounded by three loops: the Ω loop, the loop between the α-3 and α-4 helices, and a third loop opposite to the Ω loop containing the α-11 helix [31,32].

KPC, which is included in the functional group 2f, is able to hydrolyze penicillins, extended-spectrum cephalosporins, and carbapenems. It is inactivated by boronic acid derivates and not inactivated by clavulanate. Only new β-lactamase inhibitors, such as avibactam, relebactam, and vaborbactam, can inactivate KPC and restore the susceptibility to β-lactam drugs [32]. For these reasons, new β-lactams/β-lactamase inhibitors (BBLI), such as ceftazidime-avibactam (CAZ-AVI), imipenem-relebactam (IMI-REL), and meropenem-vaborbactam (MER-VAB), have been licensed by the FDA and EMA [32]. Unfortunately, clones with resistance to these BBLI have already been isolated [33,34,35].

For example, KPC strains with reduced affinity to avibactam have been reported, and one of the main resistance mechanisms conferring CAZ/AVI resistance are mutations in the Ω loop region (e.g., D179Y) [33]. The modifications in the Ω loop are also associated with the recovery of carbapenem susceptibility, and they pose an important diagnostic issue. Conventional phenotypic tests based on carbapenem hydrolysis or immunoassays are unable to identify most of these KPC variants. Moreover, their antimicrobial susceptibility pattern could wrongly be assigned to an ESBL producer [36]. Other mechanisms are also involved in the resistance to the new BBLI, for example, the decreased membrane permeability. The loss of *OmpK35* and *OmpK36* porins, in combination with enzyme hyperproduction, has been associated with high MIC values of CAZ-AVI, IMI-REL, and MER-VAB [35].

### 2.2. Class B Carbapenemases

The class B metallo-enzymes are characterized by an αß/ßα sandwich fold with one or two zinc ions in the catalytic site, which is fundamental for enzyme hydrolytic activity and located at the interface between the domains [37]. They are included in the functional group 3 of the Bush–Jacoby–Medeiros classification, and they exhibit exceptional broad-spectrum activity against β-lactams, including penicillins, cephalosporins, and carbapenems, with the exception of monobactams, such as aztreonam [20,38]. MBL are not inactivated by clinically available β-lactamase inhibitors but by metal chelators, such as ethylenediaminetetraacetic acid (EDTA) and dipicolinic acid (DPA) [20,39].

Based on the amino acid homology sequence, MBL can be divided into three subclasses: B1, B2, and B3. B1 and B3 enzymes have two *Zn^2+^* ions at the binding sites with a broader spectrum of β-lactam hydrolytic activity [20,38,40]. Commonly, the MBL family includes the GIM, SIM, SPM, IMP, NDM, and VIM enzymes [30,38].

Firstly, MBL genes were detected at the chromosomal level in Gram-positive and Gram-negative environmental and opportunistic bacteria, such as *Bacillus*, *Bacteroides*, *Aeromonas*, *Legionella*, *Pseudomonas*, *Shewanella*, and *Stenotrophomonas maltophilia* [21]. B1 members, particularly NDM and VIM, are carbapenemases of primary clinical relevance due to their β-lactamase activity and their ability to spread among bacteria on transferable elements. Among these, NDM is principally associated with Enterobacterales; on the contrary, VIM is mostly linked to non-fermenting Gram-negative bacilli (NFGNB), particularly *Pseudomonas aeruginosa* [30].

#### 2.2.1. NDM

NDM was first isolated in 2008 from a Swedish traveler to India, hence its name, New Delhi MBL [41]. Despite its recent discovery, NDM has progressively spread worldwide, although it has maintained some heterogeneity of distribution depending on the geographical region.

NDM belongs to the B1 subfamily of class B carbapenemases and Bush–Jacoby–Medeiros functional group 3a. Currently, 24 NDM variants have been recognized [30,37,42]. NDM-1, which has been isolated in more than 60 species belonging to 11 bacterial families (e.g., *Aeromonadaceae*, *Alcaligenaceae*, *Cardiobacteriaceae*, *Moraxellaceae*, *Neisseriaceae*, *Pseudomonadaceae*, *Shewanellaceae*, *Vibrionaceae*, and *Xanthomonadaceae*), is the NDM variant with the broadest host spectrum [30,37].

NDM genes have been identified both at chromosomal and extrachromosomal levels. Horizontal gene transfer is the key to the success of NDM dissemination, and it is mediated by more than 350 plasmids, of which about 20 types carry the *bla_NDM_* gene in Enterobacterales [38,42,43]. Among the latter, *IncX3* is the most common, and it has a narrow host range, being associated only with Enterobacterales [38,42,43]. Enterobacterales are the main *bla_NDM_* hosts, particularly *K. pneumoniae* and, secondly, *E. coli* and the *Enterobacter cloacae complex*. *K. pneumoniae* ST11, ST14, ST15, and ST147 are the commonest strains associated with NDM production worldwide [30]. 

NDM is a lipoprotein with a molecular mass of approximately 28 kDa [39,44]. Its secondary structure is composed of nine α-helices, 17 β-strands, and three turns [39,42]. The active site is mainly composed of three loop regions, and the *Zn^2+^* ions are located at the bottom of the pocket [44]. A characteristic of NDM, in contrast to other carbapenemases, is its position in the cell, anchored to the outer membrane of Gram-negative bacteria. NDM enzymes and *bla_NDM_* genes can be secreted through outer membrane vesicles, sharing with the neighboring bacterial cells their ability to resist β-lactams [39,45].

NDM is able to hydrolyze all beta-lactams, with the exception of monobactams (i.e., aztreonam). No clinically available β-lactamase inhibitors, such as clavulanate, tazobactam, sulbactam, avibactam, and relebactam, can inactivate NDM, making the use of the new BBLI ineffective, with the exception of the association of aztreonam/avibactam (ATM/AVI) [42,46].

#### 2.2.2. VIM

VIM was first discovered in 1997 in a carbapenem-resistant *P. aeruginosa* clinical sample from an Italian patient at the Verona University Hospital, Italy, hence its name, Verona integron-borne MBL (VIM-1) [47]. However, VIM variants have successively spread worldwide and have been reported in several geographical areas [30]. 

VIM belongs to the B1 molecular subfamily of class B carbapenemases and functional group 3a [18].

More than 40 allelic variants of VIM enzymes have been identified to date, mainly belonging to three phylogenetic clusters: VIM-1-like, VIM-2-like, and VIM-7-like.

VIM-2 was first isolated in Marseille, France, from *P. aeruginosa* and shares 90% similarity with VIM-1. The latest characterized variant is VIM-7, which has been isolated from a carbapenem-resistant *P. aeruginosa* sample from Houston, Texas. It shares a 77% identity with VIM-1 and 74% with VIM-2.

Even if *P. aeruginosa* remains the most important known reservoir of these enzymes, VIM-1-like enzymes, in particular VIM-4, have been reported in Enterobacterales, mainly *Enterobacter* spp. and *E. coli*. Sporadically, VIM variants have been detected in *Moraxellaceae* (mainly *Acinetobacter* spp.) [30]. 

The VIM-associated gene, *bla*_VIM_, is carried on a gene cassette inserted into a class 1 integron, often associated with transposons and which can be inserted on the bacterial chromosome or within plasmids [38]. Sequence identity between VIM variants ranges from ~75% to >99%; substitutions at residues 224 and 228 are a hallmark of VIM variants and are of particular interest, as these positions interact with β-lactam substrates in other MBLs [38,48].

The VIM-1 enzyme is a monomeric protein of 27.5 kDa; its structure displays the overall αβ/βα fold of the MBL superfamily, with a binuclear zinc center that forms the active site situated in a shallow groove formed by the interface of the two αβ domains. Only small differences are evident between the overall fold of VIM-1 and other structurally characterized VIM variants, mainly localized at the level of residues 60–66 and 221–241 [48].

VIM enzymes are able to confer a significant decrease in susceptibility to several beta-lactams (penicillins, cephalosporins, and carbapenems), except for monobactam (i.e., aztreonam) [30].

As other MBLs, VIM enzymes are not inhibited by β-lactamase inhibitors, such as clavulanic acid or tazobactam, but by the chelator of Zn^2+^ (i.e., EDTA) or other divalent cations [21].

#### 2.2.3. IMP

Imipenemase (IMP) was first isolated in Japan in 1988 from a clinical strain of *P. aeruginosa* [49] and characterized as a novel type of β-lactamase encoded in a conjugative plasmid that is able to hydrolyze imipenem; successively, a second report of a transferable carbapenemase in *Bacteroides fragilis* was reported [21,49]. 

IMP belongs to the B1 molecular class of carbapenemases and functional group 3a [20,28].

Even if the IMP enzyme is now widely distributed, Asia has been shown to be the predominant continent of diffusion, with the highest number in Japan (25%), followed by China (17%) and France (7%) [50].

IMP is commonly transferred between organisms, especially Gram-negative bacteria, via integron class 1 or class 3, with *P. aeruginosa* as the predominant carrier, followed by *Acinetobacter baumannii* and Enterobacterales [50].

Imipenemase is encoded by *bla*_IMP_ genes as gene cassettes, and as *bla*_VIM_ resides with other resistance genes within integron structures associated with transposons, it is able to insert onto the bacterial chromosome or within plasmids [38,50]. 

To date, about 88 variants of IMP enzyme have been reported as the result of aminoacidic substitutions distributed in different regions of the protein, divided into three major clusters. However, the overall sequence alignment shows 79.3–96.7% amino acid sequence similarity, with conserved residues in the lactam ring-catalytic site [50]. 

The IMP enzyme is an asymmetric protein of 25 kDa, and the overall enzyme scaffold is well conserved within the B1 subclass, showing the typical αβ/βα fold of the MBLs, with the two Zn^2+^ atoms at the active site [51]. 

This enzyme hydrolyzes penicillins and carbapenems, especially imipenem and meropenem, as well as third-generation cephalosporins and, interestingly, some variants that are also resistant to monobactams [50]. As with other MBLs, IMP is not inhibited by clinically available β-lactamase inhibitors, such as clavulanic acid or tazobactam, but by metal chelators (i.e., EDTA) or other divalent cations [21].

### 2.3. Class D Carbapenemases

The carbapenemases included in class D, also referred to as oxacillinases (OXA), are enzymes characterized by a serine domain structure, similar to class A carbapenemases [20]. However, differences in their active site confer a distinct affinity and specificity for β-lactam substrates. They belong to functional group 2, and they are characterized by the ability to hydrolyze oxacillin, methicillin, and cloxacillin, with slow activity against carbapenems and weak activity against extended-spectrum cephalosporins [30]. They are not inhibited by metal chelators or common clinical β-lactamase inhibitors (i.e., clavulanate, sulbactam, and tazobactam), with the exception of avibactam [30].

Class D carbapenemases can be found both at the chromosomal and extrachromosomal levels. The majority of the variants are associated with NFGNB, such as *A. baumannii complex* or *P. aeruginosa* [21,30]. Regarding Enterobacterales, OXA-48-like enzymes are the main class D enzymes. Although the principal spreading mechanism among Enterobacterales is horizontal gene transfer, which is mediated by plasmids, they are less epidemiologically widespread than other carbapenemases.

#### OXA-48-like

Considering Enterobacterales, the most clinically relevant class D carbapenemase is the OXA-48-like type. This group includes several variants of enzymes, mainly OXA-48, OXA-181, OXA-232, OXA-204, OXA-162, and OXA-244 [52]. The OXA-48 variant was isolated for the first time in Turkey in a clinical sample that was positive for *K. pneumoniae* in 2001 [53].

More than 190 plasmids harboring *bla*_OXA-48-like_ in *K. pneumoniae* have been identified; the main transposon linked to horizontal gene transfer is *Tn*1999.2 located on *IncL/M*-type conjugative plasmids [52].

Enzymes belonging to the OXA-48-like group have also been isolated from other Enterobacterales, such as *E. coli* and the *E. cloacae* complex.

Even if these carbapenemases normally produce modest increases in carbapenems’ MIC, they represent a critical therapeutic issue, particularly when the expression of *bla_OXA-48-like_* is combined with membrane impermeability and ESBL or *AmpC* production.

## 3. Epidemiology of the Principal Carbapenemases Worldwide

Carbapenemases are widespread worldwide, showing a global distribution reflecting a variability based on the type of carbapenemase, both at the continental and regional levels. In particular, the global distribution of different carbapenemase types has been observed in several countries, reflecting an endemic circulation for each geographical area. *bla*_KPCs_ are the most common class A genes circulating in Enterobacteriales in North America and Europe [54]. Among class B genes are metallo β-lactamase (MBL), including the major represented NDM, VIM, and IMP enzymes and several rare carbapenemases, such as Germany imipenemase (GIM), Tripoli metallo-β-lactamase (TMB), Kyorin hospital metallo-β-lactamase (KHM), *S. fonticola* carbapenem hydrolase (SFH), Linz metallo-β-lactamase (LMB), and Adelaide imipenemase (AIM-1) [55,56].

*K. pneumoniae* and *E. coli* account for the 90% of CPE [57]; other less frequently detected CPE are the *E. cloacae* complex, *Citrobacter* spp., and, rarely, *Salmonella*, *Shigella*, and *Proteus mirabilis* [58,59].

The data from the ATLAS global surveillance program published in 2023 highlighted that the majority of meropenem-nonsusceptible (MEM-NS) Enterobacterales collected were *K. pneumoniae* (71.5%), followed by smaller proportions of *E. cloacae* (8.7%) and *E. coli* (6.6%). The 13.2% remaining included *Providencia* spp., *S. marcescens*, *Klebsiella* spp., *Citrobacter* spp., *Enterobacter* spp., *Proteus* spp., and *Morganella morganii* [54,60].

The CHINET surveillance data from 2005 to 2022 showed *E. coli* as the most commonly isolated Gram-negative bacterium, but the rate of carbapenem resistance remained low (ranging from 0.7–2.0% for imipenem); the prevalence of carbapenem-resistant *K. pneumoniae* was 10% on average, ranging from 0.8% to 28.1% in different provinces, showing an increase from the 7.4% registered in 2016 [61].

In Africa, the results of a systematic review conducted in 2023 showed that *Klebsiella* spp. represented 72.2% of the CRE analyzed, followed by minor percentages of *E. coli* (13.5%) and *Enterobacter* (8.3%) spp. [62].

In Japan, data from an epidemiological survey on CRE conducted between 2016 and 2022 showed that *E. coli* was the most commonly identified (31.3%), followed by *K. pneumoniae* (28.0%), *E. cloacae* (18.5%), and *Klebsiella aerogenes* (6.7%) [63].

In the Western Balkans area, the most prevalent CRE was *K. pneumoniae*, followed by *E. coli*, *E. cloacae*, and *P. mirabilis*, with sporadic cases of *M. morganii*, *Providencia* spp., *Klebsiella oxytoca*, and *Citrobacter sedlakii* [64].

In a nationwide, population-based observational study conducted in Norway from 2015 to 2021, of the 389 CPE identified, the most predominant was *E. coli* (50%; 193/389), followed by *K. pneumoniae* (38%; 146/389). Other Enterobacterales species identified with a lower prevalence included *Enterobacter* spp. (6%; 23/389), *Citrobacter* spp. (3%; 10/389), the *K. pneumoniae* species complex (1.3%; 5/389), *P. mirabilis* (1%; 3/389), *Providencia stuartii* (1%; 2/389), and single isolates of *S. marcescens*, *M. morganii*, and *Kluyvera* spp [65].

The rise of carbapenemase-producing *Citrobacter* spp. as the third most common species following *K. pneumoniae* and *E. coli* was ascertained in a study by Yao and colleagues [66], in which they analyzed 512 CPE and found a 4.2% increase from 10.1% in 2017 to 14.3% in 2019 [57].

A reversal of the trend in the proportion of *K. pneumoniae* compared to other Enterobacterales among CPEs was also reported by Hussein et al. (2022) in Israel, where the proportion of CPE increased gradually, reaching 72% (97/134) in 2020, while the proportion of *K. pneumoniae* dropped from 100% of 2005 to 28% (37/ 134) in 2020. The most common Enterobacterales other than *K. pneumoniae* in Israel were *E. coli* and *Enterobacter* spp., followed by *Citrobacter* spp., *K. oxytoca*, *Raoultella* spp., *Morganella* spp., *Proteus* spp., and *Providencia* spp. [67].

In Europe, carbapenem resistance remained rare in *E. coli*, but almost one-third of EU/EEA countries reported carbapenem resistance percentages above 10% in *K. pneumoniae*. Notably, the largest increase (+2.4%) in population-weighted mean AMR percentage under EARS-Net surveillance from 2018 to 2022 occurred in carbapenem-resistant *K. pneumoniae*, resulting in a significantly increasing trend. In addition, there was a significantly increasing trend in the estimated incidence of bloodstream infections with carbapenem-resistant *K. pneumoniae*, with a 49.7% increase in 2022 compared to the baseline year, 2019 [68]. The overall percentage of carbapenem-resistant *E. coli* in 2022 in EU/EEA countries was 0.2%, with a country range of 0.0%−1.5%. The overall percentage of carbapenem-resistant *K. pneumoniae* in 2022 EU/EEA countries was 10.9%, with a country range of 0.0%−72.0%. In the United Kingdom between July 2022 and June 2023, *E. coli* was the Gram-negative bacterial species with the highest rate of carbapenemase production (34.1%), followed by *K. pneumoniae* (32.6%) and *Enterobacter* spp. (16.1%) [69].

In 2018 and 2019, among 2228 MEM-NS Enterobacterales, MBLs were identified in 36.7%; among class A, KPC was identified in 25.5%, GES was identified in less than 0.1%, and OXA-48-like were found in 24.1%. MBLs were dominant in the Asia/Pacific (APAC, 59.4%) and Africa and the Middle East (AfME, 49%), while KPC were prevalent in North America (NA, 53.6%) and Latin America (LATAM, 51.9%) and OXA-48-like carbapenemases were the most commonly reported in Europe (30%) [60].

By October 2024, 217 KPC variants were registered in the National Center for Biotechnology Information (NCBI) database and Beta-Lactamase DataBase (BLDB), of which 74 showed a narrowed spectrum against cephalosporine. The two most represented KPC variants are KPC-3 and KPC-2, with different prevalences among geographic locations, e.g., *bla*_KPC-2_ is predominant in China [61,70], whereas both *bla*KPC-3 and *bla*_KPC-2_ are predominant in the Americas and Europe [54,60].

Sporadic variants of *bla*_KPC_ have been identified in the first years after its first detection in 1996 in North Carolina [71], with a dramatic increase since 2019, mainly due to mutations in the Ω-loop bonding structure (D179N/Y variants) of *bla*_KPC-3_ and *bla*_KPC-2_ determining CAZ/AVI resistance. Compared to the wild type, KPC variants presented reduced catalytic ability to carbapenems, completely restoring carbapenem activity in some cases, meanwhile responding poorly to avibactam inhibition [29]. The majority of KPC variants conferring CAZ/AVI resistance are isolated in China, South America (Colombia, Argentina, and Brazil), Europe (Italy, Spain, and Portugal), and North America (Table 3). Regarding KPC variants, in the ATLAS global surveillance, the most prevalent was KPC-3 (43.4%); other alleles identified included KPC-31, KPC-4, KPC-46, KPC-6, and KPC-66 [60].

In 2021 in USA medical centers, 57.1% of Enterobacterales were KPC-producing, showing a diminishing trend compared to 2020 (67.5%) and 2019 (73.8%); 20.4% were MBL-producing (87.09% NDM and 12.1% IMP), with an increasing trend of 3.8% compared to 2019 and 12% compared to 2020. The OXA-48-like enzymes were identified in 8.2% of Enterobacterales, which also demonstrated a slight increase with respect to the previous years (0.3% in 2019 and 3.6% in 2020) [72]. This increasing trend in NDM producers was also shown in Israel by Hussein et al., who reported a decrease in the proportion of KPC from 94% (89/95) of all CPE in 2014 to 56% (75/134) in 2020, while the proportion of NDM and OXA-48 increased from 4% to 29% (39/134) and from 2% to 12.7% (17/134), respectively [67].

In a Taiwanese multicenter surveillance study on CRE in the period of 2012–2015, KPC-2 was the most commonly identified carbapenemase (69.8% of CPE), followed by OXA-48 (8.9%), with a sixfold increasing rate over the three years, VIM-1 (8%), KPC-17 (6.2%), and IMP-8 (5.8%). The rise of OXA-48 was also reported by Wu et al. (2024) in a single-center study that found OXA-48 in 87.05% of CPE [73,74].

According to global surveillance, among MBLs, NDM accounted for 88.4%, VIM accounted for 11.1%, and IMP accounted for 0.5%. Overall, VIM-1 (76.1%), NDM-1 (68.7%), KPC-2 (54.6%), and OXA-48 (54.3%) were the most commonly detected. Other NDM variants identified included NDM-5, NDM-7, NDM-6, NDM-9, NDM-4, NDM-16, and NDM-19. NDM-5 was isolated in a comparable proportion of NDM-1 in the APAC region [54,60]. In China, according to the CHINET and CARSS reports, the major mechanism of carbapenem resistance in carbapenem-resistant *E. coli* was the production of MBL, predominantly NDM-1 and NDM-5 [61]. The same evidence was also highlighted in the study by Li et al. on fecal carriage among adults in four provinces of China [75].

Fu et al. analyzed 320 CPE strains collected from 2016 to 2021 at a children’s hospital in Shanghai, consisting of *K. pneumoniae* in 54.7% of CPE, *E. coli* in 24.4% of CPE, and *E. cloacae* complex in 20.3% of CPE, as well as others (0.6%). They found that NDM was the most identified carbapenemase (67.6%), followed by KPC (26.4%), IMP (5.3%), and OXA-48 (0.6%). NDM-Kpn was detected in 51.8% of infants and 70.8% of neonates, while KPC-producing *K. pneumoniae* was mainly isolated from non-infants (56.3%w64.3%). In NDM-Kpn, the major ST was ST278, followed by ST15 and ST11, the latter being the most commonly detected lineage, accounting for 64.6% of KPC-producing *K. pneumoniae* [76].

NDM was also the most frequently detected carbapenemase (43.1%) in Africa, with OXA-48-like being the second main carbapenemase reported (42.9%). ST101 (mainly in Tunisia and Algeria) and ST147 (especially in Tunisia and Egypt) were most commonly reported in *K. pneumoniae*, while in *E. coli*, the most commonly detected STs were ST410, ST167, and ST38 [62].

Li et al. performed a genomic analysis of 7,731 carbapenem-resistant *E. coli* of human origin collected worldwide between 2005 and 2023 [75]. The isolates were collected from 75 countries, with the United States (17.49%, 1352/7731), China (14.88%, 1150/7731), and the United Kingdom (14.73%, 1139/7731) being the main countries represented. NDM was the most common carbapenemase (52.15%, 4032/7731), with the NDM-5 variant being the most prevalent (76.24%, 3074/4032), followed by OXA (30.09%, 2326/7731), with *bla*OXA-48 being the most common (48.84%, 1136/2326), *bla*_OXA-181_ (33.66%, 783/2326), and KPC (14.72%, 1138/7731), with KPC-2 (82.78%, 942/1138) being predominant. NDM was found in 270 known STs, with ST167 (20.41%, 823/4032) and ST410 (13.17%, 531/4032) being predominant. KPC were identified in 154 known STs, with the most common STs being ST131 (31.99%, 364/1138) and ST216 (11.16%, 127/1138). OXA-48, which was detected in 249 known STs, was most prevalently identified in ST38 (23.73%, 552/2326) and ST410 (14.4%, 335/2326). ST131 was also the most commonly identified in isolates carrying VIM (22.34%, 21/94) and IMP (41.79%, 56/134) carbapenemases. The NDM-5 variant became the most prevalent and commonly identified in China, while the majority of KPC-producing *E. coli* was reported in the United Kingdom. Although OXA-48 was reported as the most common carbapenemase in India [77], South Africa [78,79], and Russia [80], in the study by Li and colleagues, the majority of *bla*_OXA-positive_ CRECs were isolated in France in ST38 [75].

*K. pneumoniae* NDM-5 was identified in pediatric patients from 2019 to 2021 in a teaching hospital in China. Some of these were of the ST2407 group, showing the capsular serotype K25; in these isolates, theblaNDM-5 gene was carried on nonconjugative IncX3 plasmids associated with deleting the T4SS-encoding genes [57].

*E. coli bla*_NDM-5_, *bla*_NDM-1_, *bla*_NDM-4_, and *bla*_NDM-13_ were identified in inpatients in a tertiary hospital in Fujian, China, from 2021 to 2023. The co-existence of *bla*_NDM-5_ and *bla*_OXA-48_, *bla*_NDM-5_, or *bla*_NDM-4_ and *mcr-1* has been detected in few cases. *bla*_NDM-1_, *bla*_NDM-13_, and, with high frequency, *bla*_NDM-5_ exhibited conjugative results. No association between NDM variants and ST was found [81].

A study on VIM epidemiology based on two global surveillance programs analyzing data from 2008 to 2014 reported VIM-producing Enterobacterales in 17 countries, mostly in Europe (88.8%), followed by Africa (4.5%), and mainly in *K. pneumoniae* (50.6%) and *E. cloacae* (37.1%) [81,82,83]. Regarding the VIM variants, VIM-1 accounted for 75.3% and was found worldwide, followed by VIM-4 (7.9%), which was mainly identified in Europe (Czech Republic, Hungary, and Italy), Egypt, and Kuwait. Other less frequently identified alleles were VIM-2 (in Mexico and Spain); VIM-5 and VIM-31 (Turkey); VIM-19, VIM-26, VIM-27, and VIM-33 (in Greece); VIM-29 (in Saudi Arabia and the UK); and VIM-23 (in Mexico) [81,82,83]. In a recent global survey, VIM-1 remained the most frequently identified. Less frequently detected VIM variants included VIM-4, VIM-5, VIM-19, VIM-23, and VIM-24. Notably, 7.9% of the MEM-NS Enterobacterales co-carried two carbapenemases, with NDM + OXA-48-like as the most frequently identified combination, and mainly identified in the APAC; the data were also confirmed for the period 2020–2022 [54,60].

Among the major MBLs, IMP is the least frequently identified, with the exception of Japan, where it was the only carbapenemase detected in a study of 171 CRE isolated at 23 hospitals in Nara between 2018 and 2021 [84]. The main reported IMP variants in Japan are IMP-6, followed by IMP-1 and IMP-19 [84,85].

To date, 19 variants have been reported among the OXA-48 family with carbapenem-hydrolyzing activity.

According to the last report of the English Surveillance Programme for Antimicrobial Use and Resistance (ESPAUR) [69], in 2022, 3,315 Enterobacterales were confirmed as carbapenemase-positive. The “big five carbapenemase” families (KPC, OXA-48-like, NDM, VIM, and IMP) accounted for >98% of CPE, with 8% of isolates harboring more than one carbapenemase gene. OXA-48-like enzymes remained the most frequently detected carbapenemase (34.9%), followed by NDM (28.6%) and KPC (28.3%). Interestingly, from 2021 to 2022, four OXA-23-producing Enterobacterales (three *P. mirabilis* and one *E. coli*) have been identified, highlighting the limitations of rapid assays designed to target only the “big 5”.

OXA-48 was reported as the most common carbapenemase in India [77], South Africa [78,79], and Russia [80]. *E. coli bla*OXA-positive enzymes were isolated at high levels in France in ST38 [75].

In Norway, OXA-48-like enzymes accounted for 51% (198/389), NDM was the second most prevalent, with 34% (134/389), followed by KPC (6%; 23/389), VIM (2%; 8/389), and IMI/NMC-A (1%; 5/389). One GES-9 was also reported. In the 5,1% of the isolates, a dual presence of carbapenemases was found [65].

The most prevalent carbapenemase identified in the Western Balkan area was OXA-48, showing a shift in carbapenemase epidemiology from the NDM type that was prevalent in 2013–2014, although with differences in the different countries [64]. The first carbapenemases identified in Serbia in 2008 were IMP and VIM enzymes produced by *P. mirabilis* isolates [86]. In 2011, the first case of NDM-positive *K. pneumoniae* [87] was described, and a few years later, the NDM carbapenemase was considered endemic in that region, with evidence of spread in other European countries through patients previously hospitalized in the Western Balkan area [88,89]. The OXA-48-like enzymes detected in the last global carbapenemase survey [60] included OXA-232, OXA-181, OXA-244, OXA-162, OXA-163, and OXA-370. OXA-232, which is the most frequently identified variant in the APAC and LATAM, showed an increase of 36.6% in 2019. The co-production of OXA-232 with other carbapenemases is quite common, with a prevalence of NDM-5 co-existence identified in *K. pneumoniae* in Italy [90,91], Nepal [92], India [93], and Bulgaria [94].

The OXA-181 allele is common in the APAC and AfME [54,60]. The recently discovered OXA-484 is predominantly found in Europe (the UK, Germany, Ireland, and France), Switzerland, South Africa, and China in both *K. pneumoniae* (first description) and mainly in ST410 *E. coli* in the IncX3 plasmid [95]. OXA-244 has been increasingly identified in the European Union since 2013 (European Centre for Disease Prevention and Control 2021) and has been mainly reported in Norway, Germany, Poland, and Italy, mostly in *E. coli* of ST38 and ST131 [96,97,98,99,100,101].

The most clinically relevant carbapenemases are the “big five”, but there are several other less commonly reported carbapenemases. Among class A carbapenemases are Guiana extended-spectrum (GES), imipenem resistant (IMI), non–metallocarbapenemase-A (NMC-A), *S. marcescens* enzyme (SME), SFC, and French imipenemases (FRI). The results of ESPAUR surveillance, reported in 2022, showed that less than 1% of the carbapenemases were GES (0.8%; 26/3.315) and IMI (0.7%; 23/3.315) and <0.1% were FIM, SME, and DIM (ESPAUR, 2023). The proportions of VIM, IMP, FRI, GES, and IMI were less than 2% in a global study on CREC isolates [75].

The first FRI enzyme was found in 2015 in France, and to date, 12 FRI-carbapenemase variants have been reported, all in the *E. cloacae* complex, with the exception of FRI-12, which is found in *E. coli* [102].

SME enzymes belong to molecular class A and functional subgroup 2f, including hydrolyze penicillins, early cephalosporins, carbapenems, and monobactams, but not extended-spectrum cephalosporins [55]. SME have been reported only in *S. marcescens* isolates. SME-1 was first detected in England in two *S. marcescens* isolates collected in 1982, and until now, five variants, differing by one or two amino acid substitutions, have been found infrequently and sporadically in the UK, Canada, Argentina, Mexico, and Switzerland [103,104]. SME-1 has been detected in the UK and across the USA, SME-2 has been detected in Argentina, Switzerland, Canada, and the USA, SME-3 has been detected in the USA, SME-4 has been detected in Brazil, Argentina, and the USA, and SME-5 has been found in Canada [56].

## 4. Conclusions

Modern medicine relies heavily on effective antimicrobial therapy, which should take into account the increasing prevalence of antibiotic-resistant microorganisms worldwide [105]. Indeed, the clinical impact of antibiotic resistance represents an urgent threat for clinicians and also to public health in terms of increased mortality and compromised clinical outcomes. According to a report by the World Health Organization, 5 million deaths have been linked to infections caused by antibiotic-resistant bacteria [105,106,107], and treating infections with antibiotic-resistant bacteria strains healthcare resources, with an estimated economic burden of EUR 9 billion annually in Europe. In this context, antibiotic resistance represents one of the most important global health threats [106,107,108]. Among the 18 microorganisms identified by the CDC as threats to antibiotic resistance, most of them are Gram-negative bacteria. Since 2013, CPE have been classified as urgent threats by the WHO [106]. As a consequence, carbapenems have been considered the last resort in treating life-threatening infections caused by CPE. Enterobacterales develop resistance to carbapenems mainly by producing enzymes called carbapenemases, which are usually encoded by genes carried on potentially transmissible plasmids [109]. The most clinically significant carbapenemases include KPC, NDM, VIM, IMP, and OXA-48 [110]. These enzymes, which are classified as part of groups A, B, and D according to Ambler’s classification, differ in catalytic site characteristics, substrate preference, and geographic distribution [8]. The most commonly represented are KPC in Europe and the US, while NDM carbapenemases are mainly found in Southeast Asia, with some epidemiological differences at a regional level [111]. The broad spread of CPE is caused by several factors, including extensive international travel, medical tourism, and the overuse or misuse of antibiotics in human medicine, veterinary approaches, and agriculture. As a consequence, the broad spread of CPE has resulted in the endemic circulation of these microorganisms in different regions in South Asia, the Mediterranean, and different countries in Latin America. As a consequence, containment of CPE requires a multifaceted and coordinated global approach. Surveillance systems are essential to monitor the spread of CPE and to limit the emergence of new antimicrobial resistance traits, especially in high-risk areas, such as intensive care units (ICUs). Early detection using novel diagnostic tools is key to preventing the diffusion of CPE and limiting the diffusion of new traits of resistance. In this context, molecular techniques are fundamental to identifying genes related to antimicrobial resistance, as well as understanding the transmission of such traits of resistance. Different strategies have been adopted to control and limit the diffusion of CPE. Infection control measures (i.e., strict hand hygiene, contact precautions, and environmental decontamination) represent key factors in healthcare settings to limit the spread of CPE. At the same time, stewardship programs are also crucial to curb the inappropriate use of antibiotics, thereby reducing selective pressure for resistance development.

In recent years, novel molecules have been developed to combat infections caused by multi-resistant microorganisms. Among these, the combination of beta-lactams (cephalosporine or carbapenem) and beta-lactamase inhibitors represents a valuable option to counteract the infections due to CPE with limited antimicrobial options [112]. In particular, new antimicrobial combinations, such as CAZ-AVI, ceftolozane-tazobactam [C-T], MER-VAB, and IMI-REL, have been developed to limit the increasing diffusion of CPE worldwide and to treat difficult-to-treat infections due to these MDR microorganisms. At the same time, new cephalosporins, such as cefiderocol, have been introduced into the clinical armamentarium to treat infections due to MDR pathogens [110].

However, the emergence of cases of Enterobacterales resistant to these novel therapeutic molecules has been recently observed, thus reducing the clinical impact of these novel molecules.

In conclusion, contrasting the diffusion of CPE globally requires a multidisciplinary approach involving healthcare professionals, researchers, policymakers, and, in general, the correct usage of antimicrobials. In this context, the urgent need for an integrative approach should be applied to limit the diffusion of such MDR microorganisms, restore the efficacy of old antibiotics, and preserve the activity of the novel molecules. 

## Figures and Tables

**Table 1:** Genetic features of principal carbapenemase types.

	KPC	NDM	VIM	IMP	OXA-48-like
**Discovery**	USA, 1996	India, 2008	Italy, 1997	Japan, 1988	Turkey, 2001
**Ambler** **classification**	A	B	B	B	D
**Bush–Jacoby–Medeiros** **classification**	2f	3a	3a	3a	2d, 2de, 2df
**Substrate**	Penicillins Cephalosporins Monobactams Carbapenems	Penicillins Cephalosporins Carbapenems	Penicillins Cephalosporins Carbapenems	Penicillins, cephalosporins carbapenems, (monobactam)	Penicillins Carbapenems
**Inhibitors**	Boronic acid Avibactam Relebactam Vaborbactam	EDTA DPA 2-mercaptopropionic acid	EDTA DPA	EDTA DPA	Avibactam
**Plasmids**	*ColE1* *IncA/C* ***IncF*** *IncI2* *IncR* *IncX*	*ColE10* *IncA/C2* *IncB/O/K/Z* *IncC* *IncFIA* *IncFIB* *IncFIC* *IncFII* *IncFIII* *IncHI1* *IncHI2* *IncHI3* *IncI1* *IncN* *IncN1* *IncN2* *IncL/M* *IncQ1* *IncP* *IncR* *IncT* *IncX1* ***IncX3*** *IncX4* *IncY* *IncY1*	*IncA/C or IncN group*	*IncL/M*	*ColKP3* *IncC* *IncFIA(HI1)* *IncFII* *IncHI1B* ***IncL/M*** *IncR* *IncX3*
**Main** **Enterobacterales** **producers**	*K. pneumoniae (ST258, ST512, ST11)* *E. coli*	*K. pneumoniae (ST11, ST14, ST15, ST147* *E. coli (ST167, ST410, ST617)* *E. cloacae* complex	*Enterobacter* spp. *E. coli*	*Enterobacter* spp.	*K. pneumoniae E. coli* *E. cloacae* complex

**Table 2:** Activities of new antimicrobial agents against different types of carbapenemases.

Antimicrobial Agent	Type of Carbapenemase				
	KPC	OXA-48-like	NDM	VIM	IMP
Ceftolozane/tazobactam	+	+/−	−	−	−
Ceftazidime/avibactam	+	+	−	−	−
Cefiderocol	+	+	+	+	+
Meropenem/vaborbactam	+	−	−	−	−
Imipenem/relabactam	+	−	−	−	−
Plazomicin	+	+	+	+	+
Eravacycline	+	+	+	+	+
Omadacycline	−	NA	+	+	+
Cefepime/zidebactam	+	+	+	+	+
Aztreonam/avibactam	+	+			

Abbreviations: NA, not available.

**Table 3:** Classification of principal carbapenemase in Enterobacterales.

Ambler	Bush	Carbapenemases	Localization	Variants
**A**	2f	KPC	Plasmidic	>150 variants
		GES	Plasmidic	>20 variants
		IMI	Chromosomic/ plasmidic	~ 50 variants
		NMC-A	Chromosomic	-
		SME	Chromosomic	6 variants
		SFC	Chromosomic	-
	2b, 2be, 2br	SHV	Chromosomic	189 variants
		TEM	Plasmidic	243 variants
		Pen-A	Chromosomic	>40 variants
**B**	3a	NDM	Chromosomic/ plasmidic	24 variants
		VIM	Chromosomic/ plasmidic	>40 variants 3 major clusters
		IMP	Chromosomic/ plasmidic	88 variants 3 major clusters
	-	SPM	Plasmidic	-
		GIM	Chromosomic/ Plasmidic	-
		SIM	Chromosomic/ plasmidic	-
		DIM	Chromosomic/ plasmidic	-
		KHM		2 variants
		TMB		2 variants
**D**	2d, 2de, 2df	OXA-48-like	Plasmidic	>520 variants

## Data Availability

Not applicable.
